# Unique mitochondrial DNA in highly inbred feral cattle

**DOI:** 10.1016/j.mito.2012.05.003

**Published:** 2012-07

**Authors:** Gavin Hudson, Ian Wilson, Brendan I.A. Payne, Joanna Elson, David C. Samuels, Mauro Santibanez-Korev, Stephen J.G. Hall, Patrick F. Chinnery

**Affiliations:** a Institute of Genetic Medicine, Newcastle University, Newcastle upon Tyne, UK; b Vanderbilt University Medical Centre, Nashville, TN, United States; c Department of Biological Sciences, University of Lincoln, UK

**Keywords:** mtDNA, Mitochondria, Bovine

## Abstract

The Chillingham herd of wild Northumbrian cattle remains viable despite over 300 years of in-breeding and a near-homozygous nuclear genome. Here we report the complete mitochondrial DNA sequence using ultra-deep next generation sequencing. Random population sampling of ~ 10% of the extant herd identified a single mtDNA haplotype harbouring a unique bovine variant present in all other higher mammals (m.11789C/Y421H) which may contribute to their survival.

## Highlights

► The Chillingham herd of wild Northumbrian cattle remains viable despite over 300 years of in-breeding and a near-homozygous nuclear genome. ► ~ 10% of the extant population have an identical mitochondrial genome with a unique bovine variant present in all other higher mammals (m.11789C/Y421H) which may contribute to their survival.

**Fig. 1:**
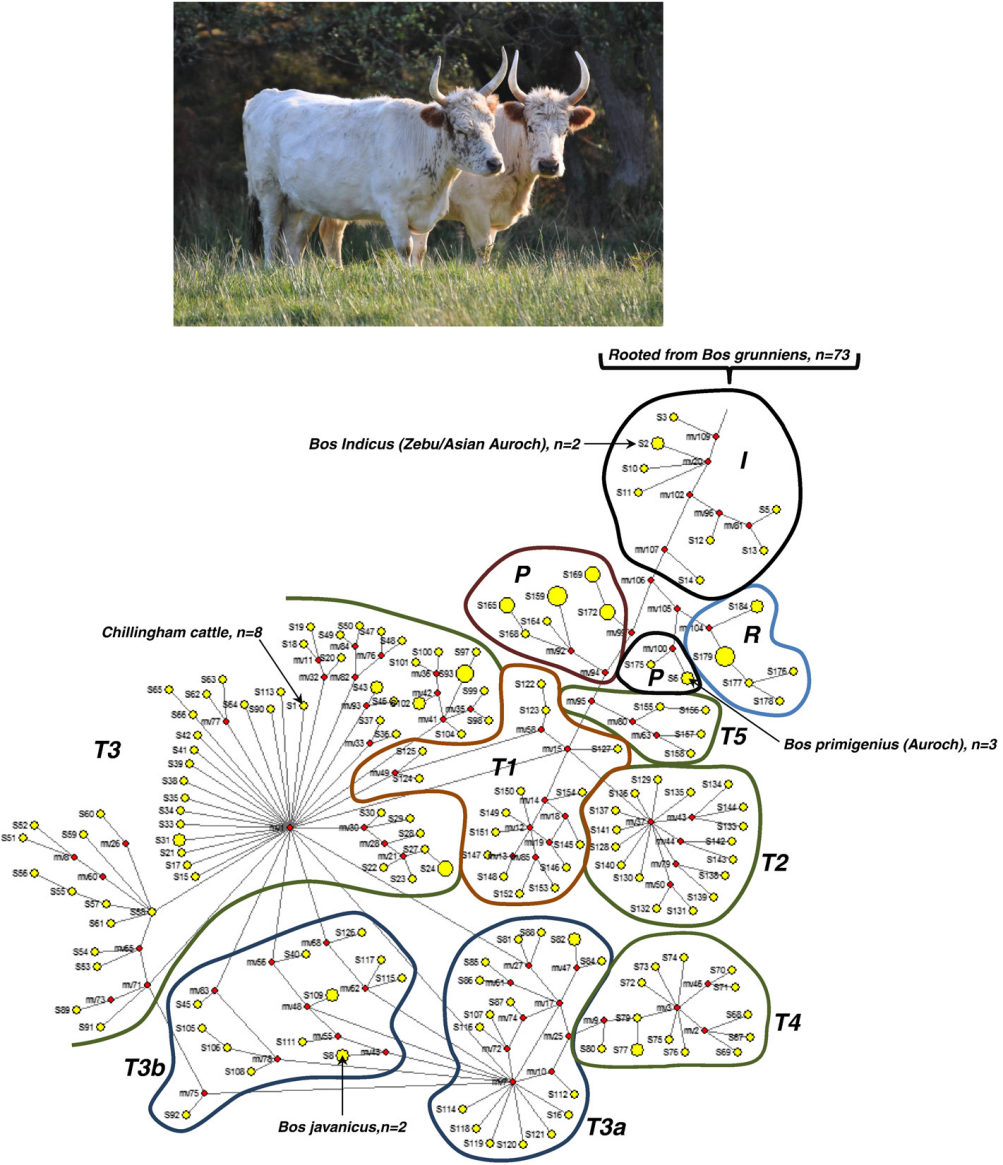
(a) The Chillingham wild cattle, *Bos taurus*. (b) Phylogenetic network of 256 complete Bovine mitochondrial DNA sequences based on coding-region variations relative to the bovine reference sequences (BRS, GenBank accession no. V00654). The relative positions and population frequencies of Chillingham cattle, Asian Auroch (*Bos indicus*), European Auroch (*Bos primigenius*) and Banteng wild cattle (*Bos javanicus*) are shown for reference. The network is shown rooted to the Yak (*Bos grunniens*) and indicates the major taurine haplogroups (Supplementary Fig. 1). Node sizes are proportional frequency and all variant weights were considered equal.

The Chillingham herd of wild cattle (Fig. 1a) has been inbred for over 300 years (67 generations), and has passed through at least one population genetic bottleneck (reduction to 5 males and 8 females in 1947). Apparently in consequence, the microsatellite genome is almost homozygous, and it is argued that the continuing viability of the herd (which now numbers 97) is due to the loss of deleterious nuclear alleles since isolation (Visscher et al., 2001). This affords a unique opportunity to study a mammalian population in the wild, where the mitochondrial genome is operating against a background of near-uniform nuclear genes.

Multiplexed next-generation sequencing, at great depth (mean coverage 2935 fold, SD = 2676), of eight randomly selected, distinct, Chillingham cattle from the extant population of 93, revealed no inter-sample sequence variation, with all carrying the same twelve mtDNA variants (m.169G; m.352G; m.2501A; m.2536A; m.2568C; m.7851C; m.8346T; m.9682C; m.11476A; m.11789C; m.13310C and 16264A), and no detectable evidence of mtDNA heteroplasmy (> 10% (He et al., 2010)). From these eight samples we can estimate that 100% of the current population has descended from a single recent female founder (Clopper–Pearson binomial 95% confidence interval = 63% to 100%).

Phylogenetic network-analysis of 256 complete mtDNA sequences, rooted with *Bos grunniens* (Yak), indicates that Chillingham cattle are related to modern cattle, and belong to the T3 sub-haplogroup (Fig. 1b). Bootstrap values indicate poor tree placement (51%, 1000 replicates, Supplementary Fig. 1), likely due to the poor resolution of haplogroup T3, which has a star-like phylogeny (Achilli et al., 2008). There was evidence of ancient extant bovine variation (Aurochs, *Bos primigenius*: m.2536A, m.9682C m.13310C, and m.16264A), inherited down the *Bos taurus* maternal lineage, and two rare variants (m.2568C and m.11476A, 5.8% and 2.9% of modern taurine mtDNAs) previously seen only in Italian cattle (Bonfiglio et al., 2010).

The Chillingham herd was stated by Darwin (1868, revised 1905) to be a “semi-wild, though much degenerated in size” descendant of the ancestor of domestic cattle, the aurochs *Bos primigenius*. Aurochs remains later than 1500 BC are not known in Britain and although there are reports of “wild cattle” from medieval Britain these were probably escapes from husbandry, and were not in districts near Chillingham. The earliest record of the Chillingham herd is dated 1646 and the most likely origin of the herd is by selection from local husbanded cattle. The idea of a connection with Roman cattle has also been advanced, but again there is no evidence that the Romans brought cattle to Britain, nor that Italian cattle were subsequently imported, so m.2568C and m.11476A are either recurrent mutations, or are more widely distributed amongst European cattle lineages.

Despite sampling ~ 10% of the extant Chillingham herd, the lack of heteroplasmy is not surprising, given the rapid shifts observed in a single maternal lineage of the Holstein cow, leading to fixation within 2 generations (Olivo et al., 1983).

All eight Chillingham cattle harboured three unique mtDNA substitutions (m.2501A in *16s rRNA*; m.8346T, a synonymous variant in *ATP6*; and m.11789C, a non-synonymous variant in *URF4*) not found in other modern taurine lineages (Supplementary Table 1). m.7851C is also found in *Bos indicus* and the modern Yak, *B. grunniens*. Given the phylogenetic relationship between these different species (Fig. 1b), m.7851C is likely to be a recurrent mutation. This is similar to other *B. taurus* breeds, which harbour 5 +/− 1.06 unique mtDNA variants (Achilli et al., 2008). Based on a phylogenetic mutation rate of 2.043 ± 0.099 × 10^− 8^/base-pair/year for the mtDNA coding region (15,247 bp) (Achilli et al., 2008), the herd is predicted to have a common maternal T3 ancestor ~ 12,000 years ago, in keeping with the Neolithic domestication of European founder cattle in the Fertile Crescent.

Inbreeding is generally found to reduce fitness in both farmed and wild animals (Visscher et al., 2001), so the continued survival of the isolated Chillingham herd suggests that deleterious alleles have been purged from the population. It is conceivable that the divergence of the Chillingham mtDNA genome contributes to the herd viability. This could, in part, be due the presence of m.11789C (Y421H), which resides in a highly conserved region of the complex I ND4 respiratory chain subunit. The histidine residue found in the Chillingham cattle is the sole allele in almost all other higher mammals (including domesticated sheep and horses), but not in modern bovine lineages (Supplementary Fig. 2), and is in a region sensitive to pathogenic mtDNA variation in humans (Taylor and Turnbull, 2005). Thus, m.11789C is likely to have a functional effect. This could occur directly through complex I activity, or indirectly though the nuclear genome, given evidence that mtDNA substitution drives the adaption in nuclear-encoded respiratory chain proteins in other species (Blier et al., 2001). Whichever is the case, since that all are healthy, the Chillingham-specific variant could optimize the aerobic synthesis of adenosine triphosphate, and thus promote herd viability in the context of an otherwise invariant nuclear genome.
