# Supplementary material for: Unique mitochondrial DNA in highly inbred feral cattle
Source: Mitochondrion. 2012 Jul;12(4):438–40. doi: 10.1016/j.mito.2012.05.003 (PMC3485552; doi:10.1016/j.mito.2012.05.003)
Supplement: Supplementary file 1 — Supplementary materials. [file mmc1.doc]

**Supporting on-line material**

**Methods**

***Next generation Sequencing***

Ultra-deep re-sequencing by synthesis (UDS) was performed by PCR amplification of complete mtDNA using 4 overlapping amplicons (Supplementary table 2). Primer specificity and miss-priming were investigated using BLAST (Altschul et al., 1997). Amplicon generation was performed in a 50μl volume containing 1× *LA* buffer for LA *Taq^TM^* DNA polymerase (Takara), 0.4 mM dNTPs, 0.3 μM primers, 1 *LA Taq^TM^* DNA polymerase (Takara) and 100ng DNA. Cycling conditions were 94 °C for 1min followed by 30 cycles of 94°C for 30s, 60°C for 60s and final extension at 72°C for 10min. Emulsion PCR and multiplexed sequencing were performed according to manufacturer's instructions (Roche 454).

***Sequence alignment***

An analysis pipeline of PyroBayes and MOSAIK (Quinlan et al., 2008; Strömberg and Wan-Ping, 2011) was used to call and align bases from the 454 flowgram output to the bovine reference sequence (GenBank accession no. V00654). Two separate alignments were performed; 1) original reference sequence and, in order to ensure that fragments overlapping at m.1 2) a rotated reference (created by displacing the first n=1000 base pairs to the end of the primary reference sequence). Subsequent analysis was based upon a consensus of these two alignments.

**Supplementary Table 1** – mtDNA variation identified in 8 unrelated Chillingham cows, showing Bovine reference (BRS - GenBank accession no. V00654), Chillingham variant (CC), its predicted amino acid change (AA) and its population frequency in taurine sequences (*Bos taurus*, modern yak - *Bos grunniens* and ancient Zebu sp. – *Bos primigenius* and *Bos Indicus*).

|  |  |  |  |  | *Bos taurus* | | *Bos grunniens* | | *Bos Primigenius* | | *Bos Indicus* | |
| --- | --- | --- | --- | --- | --- | --- | --- | --- | --- | --- | --- | --- |
| BP | BRS | CC | Locus | AA | F (n=172) | % | F (n=73) | % | F (n=3) | % | F (n=8) | % |
| 169 | A | G | D-Loop | - | 122 | 70.9 | 0 | 0.0 | 0 | 0.0 | 0 | 0.0 |
| 352 | C | G | D-Loop | - | 4 | 2.3 | 17 | 23.3 | 0 | 0.0 | 0 | 0.0 |
| 2501 | G | A | 16s rRNA | - | 0 | 0.0 | 0 | 0.0 | 0 | 0.0 | 0 | 0.0 |
| 2536 | C | A | 16s rRNA | - | 165 | 95.9 | 73 | 100.0 | 3 | 100.0 | 8 | 100.0 |
| 2568 | T | C | 16s rRNA | - | 10 | 5.8 | 1 | 1.4 | 0 | 0.0 | 0 | 0.0 |
| 7851 | T | C | CO2 | L160L | 0 | 0.0 | 73 | 100.0 | 0 | 0.0 | 8 | 100.0 |
| 8346 | C | T | ATP6 | L19L | 0 | 0.0 | 0 | 0.0 | 0 | 0.0 | 0 | 0.0 |
| 9682 | G | C | CO3 | G238A | 165 | 95.9 | 73 | 100.0 | 3 | 100.0 | 8 | 100.0 |
| 11476 | G | A | URF4 (ND4) | M316M | 5 | 2.9 | 0 | 0.0 | 0 | 0.0 | 0 | 0.0 |
| 11789 | T | C | URF4 (ND4) | Y421H | 0 | 0.0 | 0 | 0.0 | 0 | 0.0 | 0 | 0.0 |
| 13310 | A | C | URF5 (ND5) | K401T | 172 | 100.0 | 73 | 100.0 | 3 | 100.0 | 8 | 100.0 |
| 16264 | G | A | D-Loop | - | 11 | 6.4 | 73 | 100.0 | 3 | 100.0 | 0 | 0.0 |

**Supplementary Table 2** – mtDNA primary PCR primers, position in the BRS (GenBank accession no. V00654), amplicon size, melt temperature (Tm) and optimum annealing temperature (Ta).

| ID | Primer sequence (5’-3’) | Position | Amplicon | Tm(C) | Ta(C) |
| --- | --- | --- | --- | --- | --- |
| 1_F | ATGAATTACCTACGCAAGGGGTAA | -398 | 4648 | 62 | 60 |
| 1_R | ATAAACCAACATTTTCGGGGTATG | 4250 |  | 62 | 60 |
|  |  |  |  |  |  |
| 2_F | ATAAGTGGGTCCTTTACCCTCTCC | 3589 | 4692 | 62 | 60 |
| 2_R | TTTTGTTGGTGTCAGTTCTGGATT | 8257 |  | 62 | 60 |
|  |  |  |  |  |  |
| 3_F | CAGTTTCATACCCATTGTCCTTGA | 7985 | 4888 | 62 | 60 |
| 3_R | GCTACCACTATTGTGCTTGAATGG | 12872 |  | 62 | 60 |
|  |  |  |  |  |  |
| 4_F | CAAACAACCTCTTCCAGCTATTCA | 12509 | 4901 | 62 | 60 |
| 4_R | TTTCCTTTTTAGGGTTTGCTGAAG | +1071 |  | 62 | 60 |

**Supplementary Figure 1** Phylogenetic analysis of the Chillingham cow mitochondrial genome. (A) Optimal rooted Neighbour Joining (NJ) tree (Bootstrapped with 1000 replicates) detailing the relationship between all 183 complete bovine haplogroup I, P , Q, R and t mtDNA genome sequences. As in previous studies, Yak (*Bos grunniens*) and American Bison (*Bison bison*) were used as roots (Achilli et al., 2009). Evolutionary history was inferred using the Neighbour-Joining method (Saitou and Nei, 1987). (B) Maximum likelihood time scale showing the temporal relationship between the divergence of different mtDNA haplogroups (where ky = thousand years) based on the phylogenetic analysis of coding-region sequence data (364-15791).

**Supplementary Figure 2** Comparative MTND4 sequences in higher mammals showing conservation of MTND4:421H

**References for the Supplementary Material**

Achilli A, Bonfiglio S, Olivieri A, Malusa A, Pala M, Kashani BH, et al. The multifaceted origin of taurine cattle reflected by the mitochondrial genome. PLoS One 2009; 4: e5753.

Altschul SF, Madden TL, Schaffer AA, Zhang J, Zhang Z, Miller W, et al. Gapped BLAST and PSI-BLAST: a new generation of protein database search programs. Nucleic Acids Res 1997; 25: 3389-402.

Quinlan AR, Stewart DA, Stromberg MP, Marth GT. Pyrobayes: an improved base caller for SNP discovery in pyrosequences. Nat Methods 2008; 5: 179-81.

Saitou N, Nei M. The neighbor-joining method: a new method for reconstructing phylogenetic trees. Mol Biol Evol 1987; 4: 406-25.

Strömberg M, Wan-Ping L. MOSAIK, 2011.
